# Thermal stability of tungsten based metamaterial emitter under medium vacuum and inert gas conditions

**DOI:** 10.1038/s41598-020-60419-2

**Published:** 2020-02-27

**Authors:** Manohar Chirumamilla, Gnanavel Vaidhyanathan Krishnamurthy, Surya Snata Rout, Martin Ritter, Michael Störmer, Alexander Yu Petrov, Manfred Eich

**Affiliations:** 1 Institute of Optical and Electronic Materials, Hamburg University of Technology, Eissendorfer Strasse 38, Hamburg, 21073 Germany; 2 Institute of Materials Research, Helmholtz-Zentrum Geesthacht Centre for Materials and Coastal Research, Max-Planck-Strasse 1, Geesthacht, 21502 Germany; 3 Electron Microscopy Unit, Hamburg University of Technology, Eissendorfer Strasse 42, Hamburg, 21073 Germany; 4 ITMO University, 49 Kronverkskii Avenue, Saint Petersburg, 197101 Russia

**Keywords:** Nanoscale materials, Materials for optics, Nanoscale devices, Nanoscale materials, Optical materials and structures

## Abstract

Commercial deployment of thermophotovoltaics (TPV) is lacking behind the implementation of solar PV technology due to limited thermal stability of the selective emitter structures. Most of the TPV emitters demonstrated so far are designed to operate under high vacuum conditions (~10^−6^ mbar vacuum pressure), whereas under medium vacuum conditions (~10^−2^ mbar vacuum pressure), which are feasible in technical implementations of TPV, these emitters suffer from oxidation due to significant O_2_ partial pressure. In this work, the thermal stability of 1D refractory W-HfO_2_ based multilayered metamaterial emitter structure is investigated under different vacuum conditions. The impact of the O_2_ partial pressure on thermal stability of the emitters is experimentally quantified. We show that, under medium vacuum conditions, i.e. ~10^−2^ mbar vacuum pressure, the emitter shows unprecedented thermal stability up to 1300 °C when the residual O_2_ in the annealing chamber is minimized by encapsulating the annealing chamber with Ar atmosphere. This study presents a significant step in the experimental implementation of high temperature stable emitters under medium vacuum conditions, and their potential in construction of economically viable TPV systems. The high TPV efficiency, ~50% spectral efficiency for GaSb PV cell at 1300 °C, and high temperature stability make this platform well suited for technical application in next-generation TPV systems.

## Introduction

Thermal stability of the selective emitters is one of the key issues in thermophotovoltaics (TPV)^1–8^. TPV provides a viable solution for efficient heat to power conversion, offering theoretical conversion efficiencies up to 85%^2,9–16^. In TPV, a wide variety of thermal sources, such as solar heat and, waste heat from industries and, chemical and nuclear processes, can be turned into electricity using spectrally selective emitters^17–21^. Even though TPV technology has been developed many years ago, the commercial deployment of TPV is hindered due to lack of thermally stable emitter structures at temperatures higher than 1200 °C^5,22–31^. According to the Stefan-Boltzmann law^16^, the radiative power of any object is proportional to *T*^4^. Further, an ideal emitter should emit with emissivity *ε* = 1 for *E* > *E*_*g*_ (in-band photons) and *ε* = 0 for *E* < *E*_*g*_ (out-of-band photons), where *E* and *E*_*g*_ are the thermal photon energy and bandgap energy of the PV cell. Out-of-band photons will not be converted into electricity whereas these photons will be absorbed by the PV cell housing. This will increase the PV cell temperature and thereby further decrease the TPV efficiency. The out-of-band photons can be recycled using front surface filters or a mirror behind the PV cell. Front surface filters placed between the emitter and PV cell revert back the out-of-band photons to the emitter^32–34^. Alternatively, by introducing a highly reflective mirror surface at the rear of the PV cell, out-of-band photons can be directed back to reheat the emitter^29,35^. Still, the filters and mirrors have residual absorptivity which might result in a reduction of efficiency. Thus, spectrally selective TPV emitters operating at high temperatures might be required even in combination with filters and mirrors to obtain high conversion efficiency.

The highest temperature stability of structured thermal emitters of 1400 °C was achieved so far at high vacuum conditions^1,2^, of 10^−5^-10^−6^ mbar vacuum pressure, with an application of turbomolecular pumps. However, these working conditions are economically not viable in the commercialization of TPV technology. At medium vacuum condition of 10^−2^ mbar, which can be achieved without turbomolecular pump, the thermal stability of metal-based emitters drastically decreases at high temperatures^1,7,26,36^. For example, we have shown that 1D multilayered metamaterial emitters from W-HfO_2_ nanolayers can be thermally stable only up to 1000 °C under 10^−2^ mbar medium vacuum pressure^1,36^. The oxidation of refractory metals is limited only by diffusion of oxygen through the protection layers, such as HfO_2_^4,37,38^. The amount of transported oxygen is dependent on the residual O_2_ partial pressure and temperature. From our study on the stability of W-HfO_2_ multilayered metamaterial at different vacuum pressures^1^, we learned that for the same annealing time a 10-fold reduction in the residual O_2_ partial pressure can help to improve the thermal stability of the metamaterial structure by approximately 150 °C higher. Thermal stability of the emitters can be further improved by minimizing the residual O_2_ in the annealing chamber. In order to deploy TPV technology over a large scale, TPV systems should be operated under a reasonable cost and thermal emitters should sustain at high temperatures. TPV systems operating under medium vacuum and enclosed with inert gas conditions can boost the deployment of efficient TPV systems. Inert gases, such as Ar, can be used to minimize the O_2_ partial pressure in the medium vacuum.

In the current work, W and HfO_2_ materials have been used to construct 1D metamaterial emitter. W has been chosen due to its high melting point of 3422 °C, low-emissivity in the infrared region and low-vapour pressure compared to other refractory metals. Refractory oxides, nitrides, carbides and borides can be used as dielectric material to construct the metamaterial. However, borides, carbides and nitrides often have metal-like optical properties and exhibit in-band and out-of-band emission^2,34^. Refractory oxides, such as Al_2_O_3_, HfO_2_, and ZrO_2_ show dielectric properties and are stable in oxidizing atmospheres. Thermal stability of the Al_2_O_3_ based emitters demonstrated so far lies below 1300 °C^28,39–41^. ZrO_2_ suffers from phase change transition (from monoclinic to tetragonal at around 1000 °C) at high temperatures^42,43^. Whereas HfO_2_ also undergoes a similar monoclinic to tetragonal phase transition but around 1700 °C^44^, providing better phase stability at a higher temperature. Thus, HfO_2_ was chosen in the present work.

The thermal stability experiments were performed in a vacuum chamber that is enclosed in an Ar gas atmosphere. Multilayered 1D W-HfO_2_ based metamaterial structure is investigated for high temperature stability under 2×10^−2^ mbar vacuum conditions enclosed with argon gas atmosphere with 20 ppm residual oxygen. 1D metamaterial emitter shows exceptional thermal stability up to 1300 °C and to the best of our knowledge 1300 °C is the highest temperature reported for an emitter so far operating under medium vacuum condition. Presented 1D emitter exhibits a spectral TPV efficiency of 50% for GaSb PV cell with a bandgap of 0.72 eV.

## Results and Discussion

**Figure 1:**
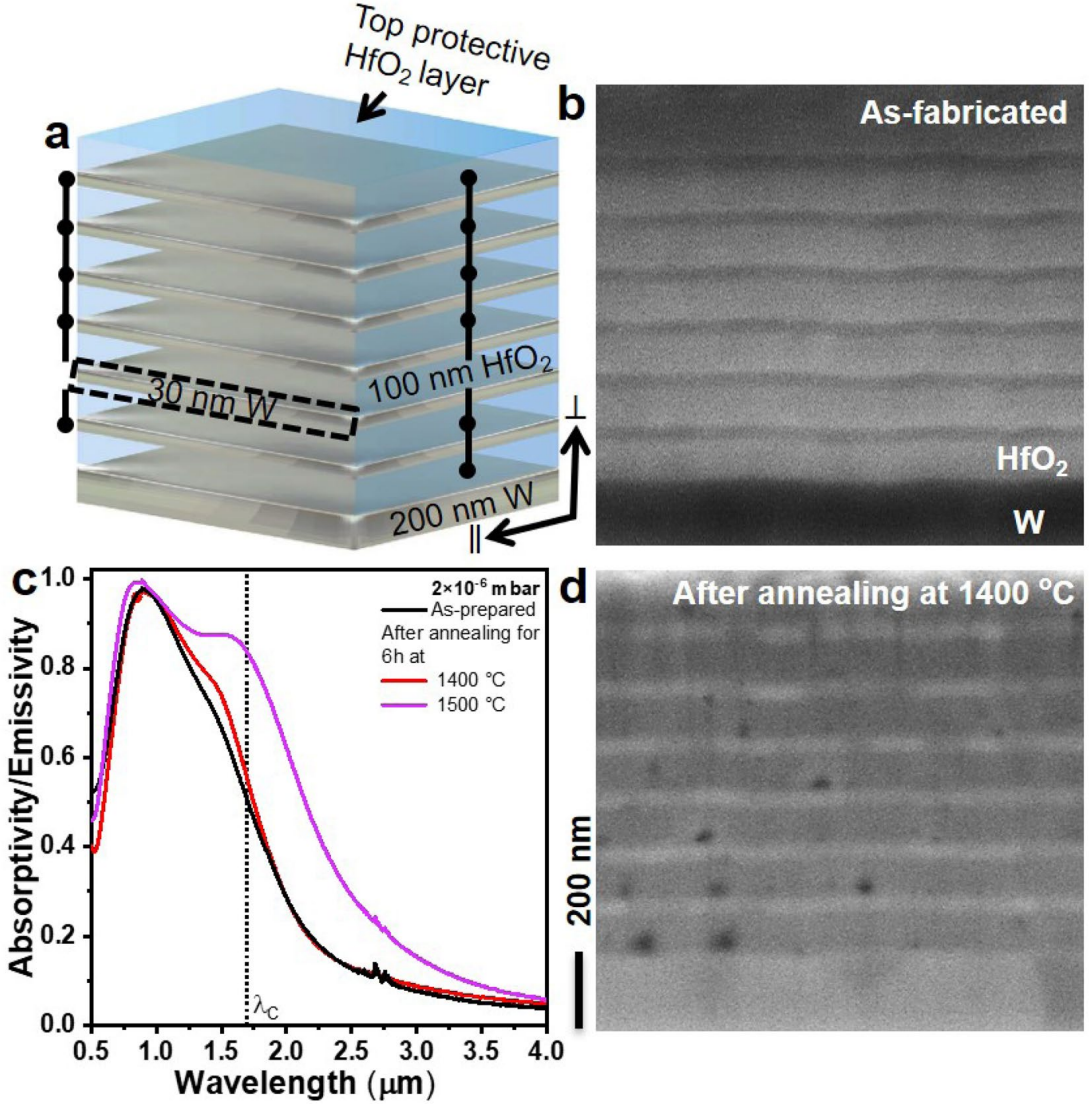
(**a**) Schematic illustration of the W-HfO_2_ emitter structure. (**b,d**) Cross-sectional SEM images of the emitter structure for as-fabricated, and after annealing at 1400 °C for 6 h under vacuum pressure of 2×10^−6^ mbar, respectively. **(c)** The measured absorptivity/emissivity spectra of the emitters shown in (**b,d**) are taken at room temperature. λ_C_ represents the cut-off wavelength at 1.72 μm, corresponding to the bandgap of the GaSb PV cell at 0.72 eV.

Figure 1a shows the schematic of the investigated W-HfO_2_ emitter structure, six bilayers of W and HfO_2_, with a thickness of 30 and 100 nm each, respectively, are sandwiched between a top protective HfO_2_ layer and bottom thick W layer with a thickness of 100 nm and 200 nm, respectively. Due to the total thickness of the metal layers in the metamaterial emitter structure, light transmission through the emitter structure can be neglected. According to Kirchhoff’s law of thermal radiation^45,46^ the emissivity of a hot radiating body equals its absorptivity. Thus, the emissivity can be directly deduced from its absorptivity. The spectral selectivity of the emitter should be maintained at different emission angles. Otherwise, an efficiency reduction can be expected due to out-of-band photons emitted at oblique angles. As we have shown previously, a 1D W-HfO_2_ metamaterial emitter shows constant spectral selectivity up to 70° incidence angles^36^, which is one of the main advantages of metamaterial emitters in comparison to resonance-based emitters^11^.

Figure 1b shows the cross-sectional scanning electron microscope (SEM) image of the as-fabricated emitter structure, where the distinction between HfO_2_ and W layers can be clearly seen with a smooth interfacial surface. The measured absorptivity/emissivity spectrum of the as-fabricated emitter structure is shown in Fig. 1c-black trace. The metamaterial is designed to operate at high temperatures up to 1400 °C. In accordance with the blackbody radiation at 1400 °C, the band-edge spectral characteristics were tailored to obtain high and low emissivities below and above, respectively, the spectral-cutoff around 1.72 μm of GaSb PV cell. Based on the previous works^1,36^, we have optimized the metal and dielectric layer thickness values in order to minimize the residual absorption in the mid-infrared region. A 30 nm thin film based metamaterial (Fig. 1c) shows around 2× less mid-infrared absorptivity/emissivity compared to a 20 nm thin film metamaterial emitter^1,36^. Thermal photons coming from a radiative blackbody span over a wide range of wavelengths, where most of the energy is residing in the long-wavelength regime. Thus, reducing the emissivity in the long-wavelength region will avoid heating of the PV cell housing package and thus help to maintain the external quantum efficiency of the PV cell^47^ without the use of additional front surface filters, which would impose another restriction on the thermal stability^32–34^. The reduced absorptivity/emissivity in mid-infrared range with larger W thickness can be attributed to the change in electron collision frequency of the metal films, where it decreases with an increment in film thickness. This contributes to the reduction in the imaginary part of the dielectric function ε″ in the mid-infrared range, thus improving the reflectivity of the metal^48,49^. The absorptivity/emissivity spectrum clearly shows a step function-like band-edge spectral characteristics around 1.72 μm, where high/low absorptivities/emissivities, respectively, are absorbed below and above the wavelength corresponding to the cut-off wavelength at 1.72 μm of the PV cell. In the mid-infrared region, the absorptivity/emissivity is around 7%, which is close to the absorptivity/emissivity of the bulk W metal.

**Figure 2:**
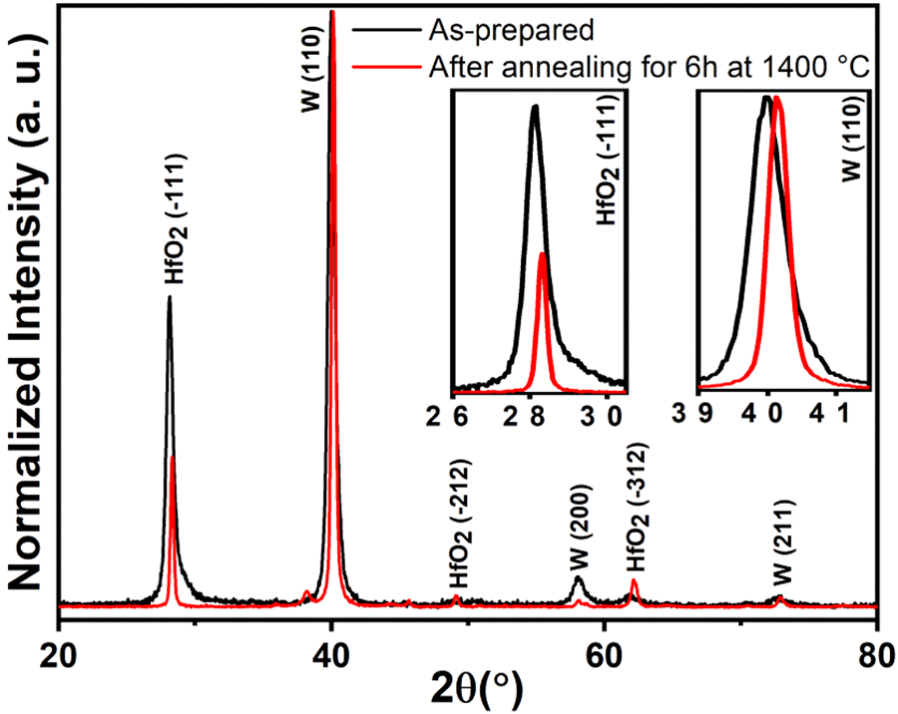
XRD spectra of the emitter before and after annealing at 1400 °C for 6 h under vacuum pressure of 2×10^−6^ mbar. XRD spectra were normalized with respect to dominant (110) peak of bcc W. Insets show the magnified view of HfO_2_ and W reflexes for (−111) and (110) planes, respectively.

Figure 2 shows the XRD analysis of the metamaterial structure before and after annealing at 1400 °C for 6 h under vacuum pressure of 2×10^−6^ mbar, where both, as-prepared and heat-treated, structures exhibit polycrystalline nature. W has the bcc structure (JCPDS 00-004-0806) of α-phase with (110), (200) and (211) planes at 2θ = 40.3°, 58.3° and 73.2°, respectively, and HfO_2_ has a monoclinic structure (JCPDS 034-0104), with (-111), (-212) and (-312) planes at 2θ = 28.3°, 49.1° and 62.4°, respectively. After annealing at 1400 °C for 6 h, the diffraction peaks of W and HfO_2_ were narrowed, which confirms the increment in W and HfO_2_ grain sizes. W and HfO_2_ grain sizes were calculated using the Scherrer formula^50,51^ taking into account the (110) and (-111) reflexes, respectively. For the as-prepared structure, W and HfO_2_ show grain sizes of 13 and 12 nm, whereas after annealing at 1400 °C grain sizes were increased to 21 and 27 nm, respectively.

**Figure 3:**
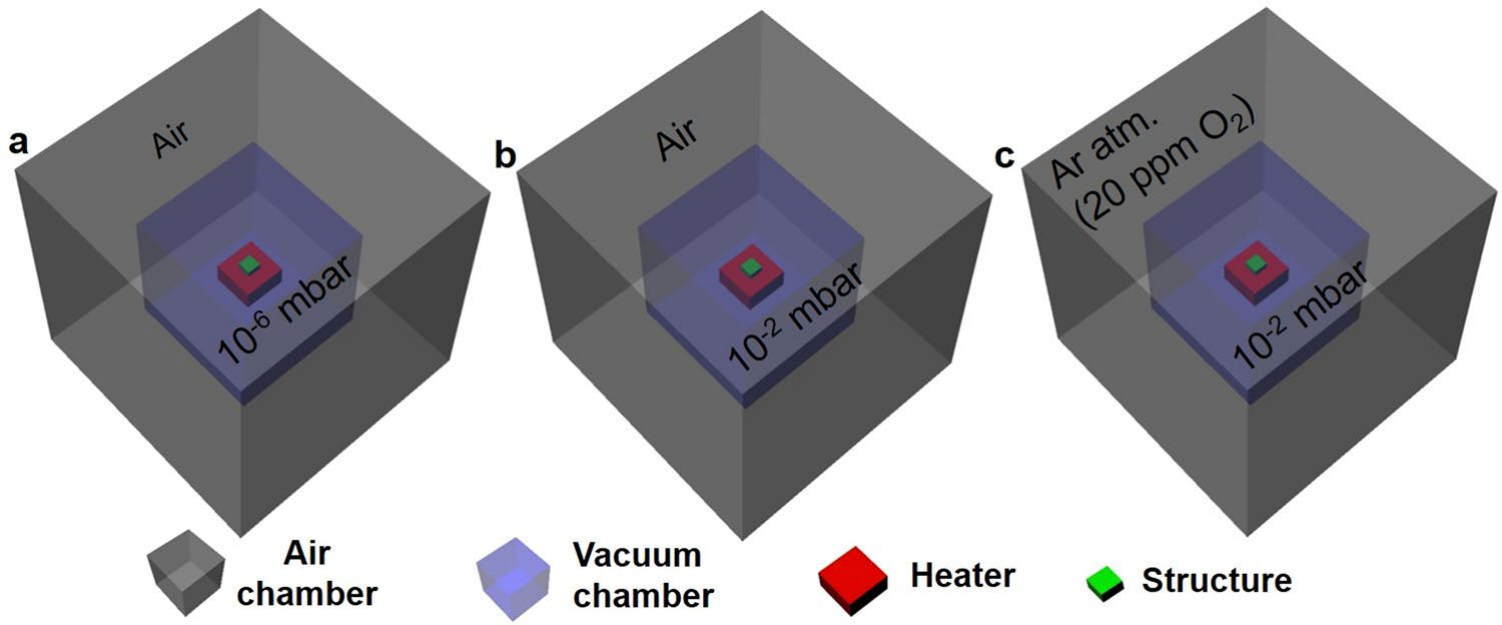
Schematic presentation the annealing schemes used to investigate the thermal stability of the emitter structure. Objects are not in scale.

Figure 3 shows the annealing schemes used in the present work. In Fig. 3a, the thermal stability of the metamaterial emitter is investigated using a high vacuum chamber oven operating at 2×10^−6^ mbar vacuum pressure surrounded by ambient air atmosphere, whereas in Fig. 3b the emitter is annealed in a medium vacuum chamber oven operating at 2×10^−2^ mbar vacuum pressure surrounded by ambient air atmosphere. In the schematic shown in Fig. 3c, a medium vacuum chamber oven operating at 2×10^−2^ mbar vacuum pressure encapsulated by Ar atmosphere is used for annealing experiments. As a first step, we have annealed the metamaterial structures in a high vacuum chamber as shown in Fig. 3a. The emitter structure is subjected to the annealing temperature of 1500 °C for 6 h under 2×10^−6^ mbar vacuum pressure. The measured absorptivity/emissivity spectra taken at room temperature after annealing at 1400 °C is shown in Fig. 1c-red trace, which is almost identical to the spectral features observed in the as-fabricated emitter structure. The corresponding cross-sectional SEM image of the emitter structure is shown in Fig. 1d. The W layers show rough surfaces due to grain growth at high temperatures, and voids in the HfO_2_ layers owing to grain growth (Fig. 2) through intra-layer diffusion of HfO_2_. Except for these minor structural changes, the metamaterial emitter structure shows exceptional spectral stability and structural integrity after annealing at 1400 °C for 6 h under 2 × 10^−6^ mbar vacuum pressure. The O_2_ partial pressure in the vacuum chamber is estimated to be in the order of 4 × 10^−7^ mbar (assumed to be 21% of vacuum pressure coming as leakage into vacuum from air environment)^52^. No traces of W oxidization is seen, any formed volatile tungsten oxides sublimate immediately and leave voids in the W layers at these temperatures^1^. At 1500 °C, the metamaterial emitter structure shows spectral degradation, i.e., high emissivity in the long-wavelength region due to the structural changes in the emitter structure, owing to grain growth and void formation in HfO_2_ layer. Under high vacuum conditions, the thermal stability of the 1D metamaterial emitter is limited by the intrinsic structural changes in W and HfO_2_ layer.

**Figure 4:**
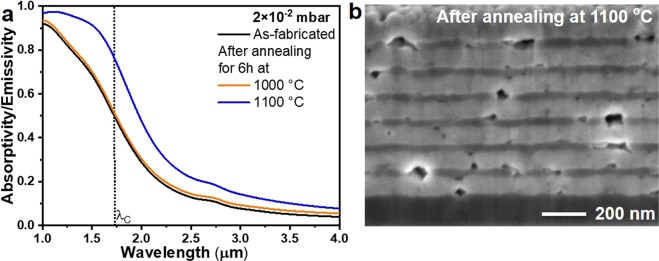
(**a**) Absorptivity/emissivity spectra of the emitter, taken at room temperature, for as-fabricated, and after annealing at 1000 °C and 1100 °C for 6 h under medium vacuum pressure of 2×10^−2^ mbar. (**b)** Cross-sectional SEM image of the emitter structure after annealing at 1100 °C for 6 h under 2×10^−2^ mbar vacuum pressure.

In order to study the influence of medium vacuum condition on the spectral/structural stability of the 1D metamaterial structure, the emitter structures are annealed under 2×10^−2^ mbar vacuum pressure, showed in Fig. 3b. The measured absorptivity/emissivity spectra of the metamaterial emitter taken at room temperature for as-fabricated and, after annealing at 1000 and 1100 °C for 6 h under 2 × 10^−2^ mbar vacuum pressure is shown in Fig. 4a. No change in the optical spectrum of the emitter is observed after annealing at 1000 °C (Fig. 4a-orange trace), whereas spectral degradation, an increase of absorptivity/emissivity throughout the spectral region, is clearly observed after annealing at 1100 °C (Fig. 4a-blue trace). The residual O_2_ partial pressure in the vacuum chamber is in the order of 4 × 10^−3^ mbar. The top protective HfO_2_ layer limits oxygen diffusion for 6 h at 1000 °C. W and HfO_2_ films show nanocrystalline nature (Fig. 2) and (usually nanocrystalline materials contain grain boundaries) offer various diffusion channels. Since a diffusion process is dependent on temperature, we observe that for the same time at 1100 °C progression of oxygen diffusion from the external environment through HfO_2_ layer to the W layers is oxidizing the W surfaces^1,36^. The cross-sectional SEM image of the metamaterial emitter structure after annealing at 1100 °C is shown in Fig. 4b, where voids are clearly seen in W and HfO_2_ layers. Oxygen can diffuse in atomic form through HfO_2_ by interstitial or exchange process^53,54^. This might alter the HfO_2_ crystal structure, promote grain growth and lead to the formation of voids in the HfO_2_ layer. Oxygen diffusion through HfO_2_ and transportation via voids oxidizes the W and creates holes in W layer due to sublimation of volatile W oxides^55,56^. The grain growth of HfO_2_ can also rupture the W layers^1^.

Thus, metamaterial emitter structures show a thermal stability limit of 1000 °C under medium vacuum condition of 2 × 10^−2^ mbar vacuum pressure, which can be improved further by reducing the residual O_2_ content in the annealing chamber.

**Figure 5:**
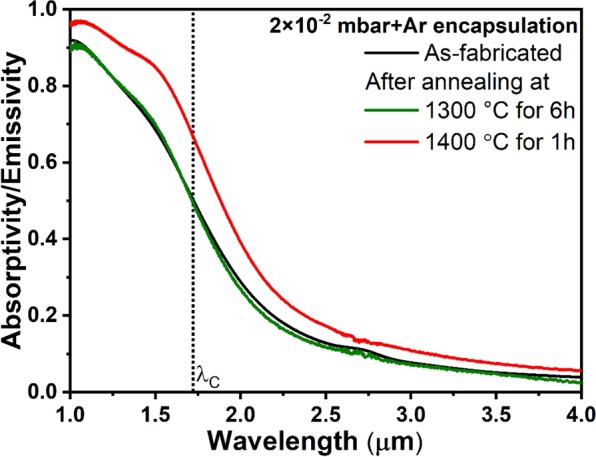
Experimental spectral absorptivity/emissivity of the emitter structure measured at room temperature for as-fabricated and after annealing at 1300 °C for 6 h and 1400 °C for 1 h under 2 × 10^−2^ mbar vacuum pressure and encapsulating the vacuum chamber with Ar atmosphere.

**Figure 6:**
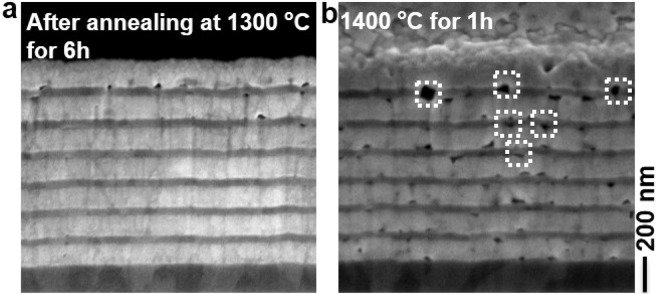
Cross-sectional SEM image of the emitter structures after annealing at 1300 °C for 6 h **(a)** and 1400 °C for 1 h **(b)** under 2×10^−2^ mbar vacuum pressure and encapsulated by the Ar atmosphere. Note that a thin Au/C layer is sputter deposited on the sample prior to the FIB milling to prevent charging of the substrate during imaging. White dotted boxes indicate visible cross-sectional areas of voids formed after annealing.

In order to minimize the residual O_2_ content in the medium vacuum condition, we have encapsulated the annealing chamber with Ar atmosphere, Fig. 3c. The purged argon has the purity of 99.999%, due to residual air in the encapsulation region we have measured the O_2_ content in the encapsulated area of 20 ppm (see experimental section). Thus, the effective O_2_ partial pressure in the annealing chamber is dramatically reduced from ~10^−3^ to 10^−7^ mbar. Thermal stability of the 1D metamaterial emitter structure is investigated by annealing the emitter structures up to 1400 °C, and the corresponding typical optical spectra after annealing at 1300 and 1400 °C are shown in Fig. 5. After annealing at 1300 °C for 6 h, the optical spectrum shows no variance compared to the as-fabricated structure and the corresponding cross-sectional SEM image of the emitter is shown in Fig. 6a. No visible damage to the W layers is observed, such as voids due to sublimation of W oxides. However, HfO_2_ layers still show voids, particularly in the top layers, but the number of voids observed in the structure is much less than in the case observed for medium vacuum conditions without Ar gas encapsulation (Figs. 3b and 4b). The reason is the decrease of O_2_ partial pressure in the annealing chamber due to Ar gas encapsulation, and thereby reduction of the oxygen transfer through HfO_2_ and subsequent structural changes. When the metamaterial emitter is annealed at 1400 °C for 1 h, the optical spectrum shows higher absorptivity/emissivity below and above the λ_c_ (Fig. 5-red trace), i.e. spectral degradation. A typical cross-sectional SEM image of the metamaterial structure after annealing at 1400 °C is shown in Fig. 6b, which clearly shows the voids in HfO_2_ as well as W layers (white-dotted rectangles). We have taken multiple cross-sectional images and found that the visible combined cross-sectional area of voids formed in the top thin W layers is higher than in the lower thin W layers, whereas the bottom W layers are left largely intact (Fig. 6b). Since, oxygen is diffusing from the external environment to the emitter, a 100 nm top protective HfO_2_ layer successfully prevents residual oxygen diffusion up to 1300 °C for 6 h, whereas at 1400 °C, according to the temperature enhanced oxygen diffusion, larger voids are formed randomly in the W layers already after 1 h annealing due to subsequent sublimation of volatile W oxides.

**Figure 7:**
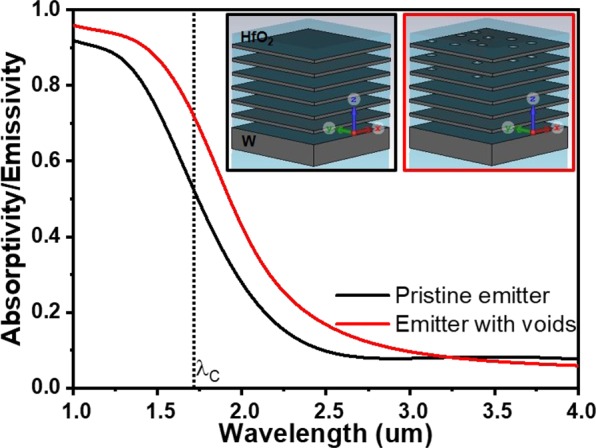
Calculated absorptivity/emissivity spectra of the emitter structure for pristine emitter and emitter with voids, the corresponding unit cell schematics are shown in the inset.

Thus, oxygen transport through HfO_2_ is governed by partial oxygen pressure and temperature, which leads to grain growth in HfO_2_ and formation of voids in the layer. This, in turn, leads to a rupture of the W layers next to the HfO_2_ layers and to voids as observed in the sample after annealing at 1100 °C without encapsulation. Some of the tungsten atoms in the upper layers oxidize and leaves the structure due to sublimation of volatile W oxides. The results of annealing at 1300 °C and 1400 °C test can be interpreted as an accelerated test. We see that the encapsulated structure degrades at 1300 °C for 6 h to a smaller degree than at 1400 °C for 1 h. In fact, there is at least a 10 times durability improvement per 100 °C. With that, our emitter should operate more than 1000 h at 1100 °C in an encapsulated vacuum chamber. The exact determination of the scaling factor will be the goals of our future investigations. In order to understand the change in the absorptivity/emissivity of the emitter after annealing at 1400 °C, we have calculated absorptivity/emissivity spectra of the emitter structure for pristine emitter and emitter with voids. The calculated absorptivity/emissivity spectra of the emitter structure without any structural changes (pristine emitter, Fig. 7-black trace) shows high and low emissivities below and above the cut-off wavelength, respectively, similar to the as-fabricated structure shown in Fig. 5. When the voids are implemented as holes in the top three 30 nm W layers of the emitter (schematic of the unit cell is shown in the inset of Fig. 7), the calculated absorptivity/emissivity spectrum shows a red shift in spectral profile and higher emissivities below and above the cut-off wavelength position compared to pristine emitter. The red shift of the spectrum is expected and can be explained by the decrease of the total amount of W content in the thin layers. According to the effective medium approximation^36,57^ the effective permittivity will change due to vacuum holes shifting the near zero epsilon point to longer wavelengths.

Thus, under medium vacuum condition with reduced residual O_2_ content, the thermal stability limit of the 1D metamaterial emitter is increased to 1300 °C. Reducing further the residual oxygen content in the encapsulated area, the thermal stability of the emitters can be matched with the case of high vacuum conditions, up to 1400 °C.

**Figure 8:**
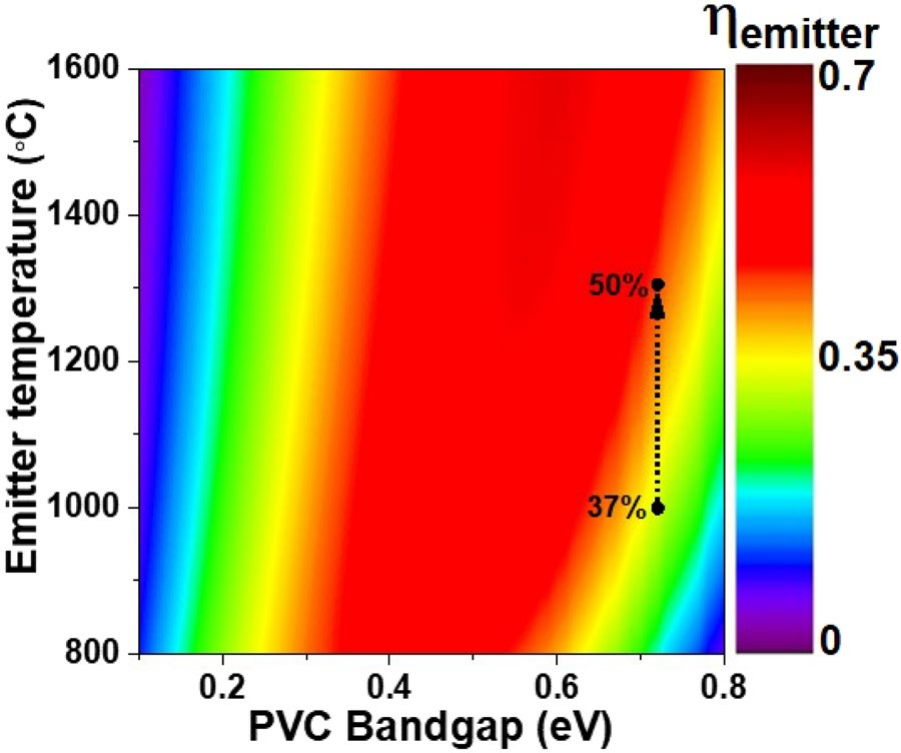
The TPV conversion efficiency of 1D metamaterial emitter structure. Contour map shows the emitter efficiency *η*_*emitter*_ of 1D layered metamaterial emitter structure for temperature versus PV cell bandgap energy.

The spectral efficiency of the emitter, *η*_*emitter*_, is calculated using Eq. 1.

1 $${\eta }_{emitter}={\int }_{{E}_{g}\,}^{\infty }\frac{{E}_{g}}{E}\varepsilon (E){I}_{BB}(E,{T}_{emitter})dE/{\int }_{0}^{\infty }\varepsilon (E){I}_{BB}(E,{T}_{emitter})dE$$

where *ε*, *E*, *T*_*emitter*_, and *I*_*BB*_ correspond to the spectral emissivity, photon energy, emitter temperature and blackbody spectral power density at the emitter temperature, respectively. Figure 8 shows the *η*_*emitter*_ with respect to temperature and PV cell bandgap. When the metamaterial emitter is operating under medium vacuum conditions and the annealing chamber is encapsulated in Ar atmosphere, it shows 37% efficiency at 1000 °C for PV cell bandgap of *E*_*g*_ = 0.72 eV (GaSb PV cell). When the temperature is raised to 1300 °C the emitter shows 50% spectral efficiency. If a blackbody radiator is used instead of the metamaterial emitter, it will show only 16% conversion efficiency at 1300 °C. Thus, 1D multilayered metamaterial emitter exhibits 3.1× higher efficiency than a blackbody operating at 1300 °C. It should be mentioned that calculated efficiency is based on spectral absorptivity measured at normal incidence. But due to weak angle dependence of absorptivity/emissivity as discussed earlier, we expect that this efficiency holds also for wide angle emission, up to ±70°^36^.

## Conclusion

In summary, we have investigated the effect of residual O_2_ partial pressure on the thermal stability of the emitter structures at high temperatures. Under high vacuum conditions, ~10^−6^ mbar vacuum pressure, the metamaterial emitter shows unprecedented thermal stability up to 1400 °C. At temperatures higher than 1400 °C, the thermal stability of the emitter structure is limited by the structural changes in HfO_2_ and the oxidation does not play any role in the emitter degradation. Under medium vacuum conditions, ~10^−2^ mbar vacuum pressure, the emitter structure shows thermal stability up to 1000 °C, and at higher temperatures W and HfO_2_ layers in the emitter structure show degradation due to oxidation. By encapsulating the annealing chamber operating under medium vacuum condition with Ar atmosphere, the metamaterial emitter structure shows outstanding spectral/structural stability of 6 hours at a temperature of 1300 °C. The cumulative effect of medium vacuum condition and inert gas encapsulation improves the thermal stability limit of 1D emitters to 1300 °C, which paves the way towards building the next generation TPV systems.

## Methods

### 1D metamaterial emitter structure fabrication

Metamaterial emitters with W and HfO_2_ thin films were fabricated on 5 × 5 mm^2^ single crystalline sapphire substrates ([1–102] orientation) by radio frequency and direct current magnetron sputtering at a rate of 0.2 and 0.09 nm s^−1^, respectively. All the layers are grown sequentially at argon (99.99999%) gas pressure of 2 × 10^−3^ mbar. The W and HfO_2_ sputtering targets with 99.95% purity were purchased from Sindlhauser Materials. Cross-sectional SEM images of the emitter structures were prepared using a focused-ion beam (FIB, FEI Helios G3 UC) operating at 30 keV. The secondary electron images were taken under low kV (2 kV) and high-resolution immersion mode using a through lens detector (TLD).

### Thermal annealing

Emitter structures were annealed in a high-temperature heating stage (Linkam, TS1500) at 2 × 10^−2^ mbar vacuum pressure using a rough vacuum pump, and in a high-temperature vacuum furnace (RD-G WEBB) at 2 × 10^−6^ mbar vacuum pressure. The temperature was ramped at a rate of 10 °C min^−1^. O_2_ concentration in the encapsulated area is measured using an O_2_ sensor (Sensor type: SO-B0-010, in the range up to 2000 ppm, purchased from SENSORE Electronic GmbH) and it is assembled on a generic sensor board. Ar gas (99.999% from Linde) at a pressure of 3 bar is used to encapsulate the annealing chamber.

### Absorptivity/emissivity measurements

Absorptivity/emissivity spectra of the emitter structures, for as-fabricated and after annealing at high-temperatures, were obtained by measuring reflectivity spectra using a UV-Vis-NIR spectrometer (PerkinElmer Lambda 1050) and a Fourier transform infrared spectrometer (FTIR-Vertex 70, Bruker), in the ranges of 0.3 to 2.5 μm and 1 to 5 μm, respectively. Where, absorptivity α = 1 *– ρ – τ*, where *ρ* and *τ* are reflectivity and transmissivity. A 200 nm thick bottom W layer in the emitter inhibits the light transmission through the emitter, thus absorptivity can be directly obtained from the reflectivity using α = 1 *– ρ*.

### Numerical modelling

Absorptivity/emissivity spectra of the emitter structure for pristine emitter and emitter with voids were calculated using the finite-integration-time-domain method in CST microwave studio. Optical constants of W were taken from Palik data^58^. A unit cell with 517.5 × 517.5 nm^2^ is constructed to match with the cross-sectional SEM image, and we have implemented 10, 8 and 4 holes with 100 nm diameter in the top three thin W layers, respectively. The unit cell is excited with a plane wave at normal incidence. A fine grid of 40 points per wavelength was chosen to ensure that all results were converging.

### XRD measurements

XRD measurements were taken using a Bruker D8 Advance diffractometer. Cu Kα (λ = 0.15405 nm) radiation with parallel beam geometry was used to characterize the metamaterial structure. The diffraction patterns (2θ from 20° to 80°) were recorded with an increment of 0.04° and a step time of 16 s.
